# Relationship of Liver X Receptors α and Endoglin Levels in Serum and Placenta with Preeclampsia

**DOI:** 10.1371/journal.pone.0163742

**Published:** 2016-10-13

**Authors:** Jing Wang, Xing Dong, Hong-yan Wu, Nan Wu, Xue-jun Zhang, Xin Wang, Li-xin Shang

**Affiliations:** 1 Department of Obstetrics and Gynecology, General Hospital of Beijing Military Command, Beijing, China; 2 Department of General Surgery, General Hospital of Beijing Military Command, Beijing, China; Colorado State University, UNITED STATES

## Abstract

**Background:**

Liver X receptor alpha (LXRα) and endoglin have been postulated to play roles in trophoblast invasion and lipid metabolic disturbances. However, the relationship between LXRα and endoglin levels in serum and placenta of patients with preeclampsia remains poorly understood. The objective of this study was to identify correlations between LXRα, endoglin and preeclampsia and provide new feasible methods of clinical prediction and treatment for preeclampsia.

**Methods:**

We enrolled 45 patients with preeclampsia (24 with moderate preeclampsia and 21 with severe preeclampsia) and 15 normal pregnant women (control group) who were admitted to the Department of Obstetrics of the General Hospital of Beijing Command between October 2012 and July 2013 in this study. Serum and placental LXRα and endoglin levels were analyzed by enzyme-linked immunosorbent assay, real-time quantitative PCR, tissue microarray and immunohistochemistry.

**Results:**

Serum and placental LXRα and endoglin levels were significantly higher in patients with preeclampsia than those in control group (P<0.05, each). Moreover, patients with severe preeclampsia displayed significantly higher LXRα and endoglin levels than those with moderate preeclampsia (P<0.05, each). The LXRα sensitivity, specificity and positive and negative predictive values were 66.00%, 80.00%, 89.19% and 48.48%, respectively, while those of endoglin levels were 62.00%, 85.00%, 91.18% and 47.22%, respectively. LXRα and endoglin levels in serum and placenta from patients with preeclampsia were positively correlated (serum: r = 0.486, P<0.01; placenta: r = 0.569, P<0.01).

**Conclusions:**

Elevated LXRα and endoglin levels may be associated with preeclampsia pathogenesis and development and could be used as potential predictors for this disorder.

## Introduction

Preeclampsia, a disorder unique to pregnancy, is a leading cause of maternal and perinatal mortality and morbidity with incompletely elucidated pathogenesis [1]. However, disease treatment is limited to symptomatic treatment, and individuals with severe preeclampsia may require pregnancy termination to avoid deterioration of the condition. Inadequate trophoblast invasion and uterine spiral artery remodeling leading to poor placental perfusion and hypoxia have been proposed to be involved in preeclampsia [2,3]. Additionally, lipid metabolic disturbances resulting in endothelial dysfunction trigger preeclampsia [4]. Recent observations suggested that abnormal liver X receptor alpha (LXRα) and endoglin expression, associated with lipid metabolic disturbances and trophoblast proliferation, might play important roles in preeclampsia development [4,5].

LXRα belongs to the nuclear receptor superfamily and binds to and is activated by naturally occurring oxidized forms of cholesterol, known as oxysterols [6]. It plays a key role in cholesterol metabolism. To date, the role of LXRα in preeclampsia is largely uncharacterized. Some studies have found that LXRα inhibited trophoblast invasion, human chorionic gonadotropin secretion, cholesterol transport and high-density lipoprotein biosynthesis [7]. Thus, LXRα may play an important role in the occurrence and development of preeclampsia.

Endoglin is a trans-membrane glycoprotein that serves as a co-receptor for transforming growth factor (TGF)-β1 and TGF-β3 [8]. It is expressed on placental syncytiotrophoblasts and invasive cytotrophoblasts and participates in the regulation of placental trophoblast differentiation and invasion of the uterus during pregnancy. One study found that inhibition of endoglin translation in a human extravillous trophoblast cell line could improve the capacity of extravillous trophoblast invasion and spiral artery remodeling [9]. On the other hand, endoglin is also expressed on vascular endothelial cells, which are important not only in angiogenesis but also in maintaining a healthy blood vessel lining. If the blood vessel lining is disrupted, blood pressure increases, and excess protein may be excreted into the urine.

Henry-Berger et al. demonstrated that endoglin is a direct target of LXRα in human syncytiotrophoblast cells [10]. Treatment of human choriocarcinoma JAR cells with T0901317, a synthetic LXR-selective agonist, led to a significant increase in endoglin mRNA and protein levels. On the other hand, one study demonstrated that LXR (as a heterodimer with the retinoid X receptor) is able to bind the endoglin promoter on an LXR response element and mediates the activation of endoglin gene, thus affecting proliferation, migration and invasiveness of normal human trophoblast cells. To date, the correlation between LXRα and endoglin has been studied only *in vitro*.

The aims of this study were (1) to test whether there was a relationship between LXRα and endoglin levels and preeclampsia occurrence and development; and (2) to define the relationship between LXRα and endoglin. The ultimate goal was to provide the theoretical basis for the early diagnosis, prevention and treatment of preeclampsia.

## Materials and Methods

### Ethics Statement

The study was approved by the clinical research ethical committee of the General Hospital of Beijing Military Command, and informed written consent was obtained from all subjects.

### Study population

We selected 45 patients with preeclampsia (24 individuals with moderate and 21 with severe preeclampsia) from the Obstetrics Department of our hospital between October 2012 and July 2013. Individuals with systolic blood pressure ≥140 mmHg or diastolic blood pressure ≥90 mmHg and proteinuria ≥1+ (30 mg/dl) or ≥0.3 g/24 h by dipstick testing at gestational week 20 were defined as having moderate preeclampsia. Individuals with systolic blood pressure ≥160 mmHg or diastolic blood pressure ≥110 mmHg and proteinuria ≥3+ (300 mg/dl) or ≥3.5 g/24 h or with HELLP syndrome after 20 weeks of gestation were defined as having severe preeclampsia [11]. Additionally, 15 late pregnant women with normal blood pressure were randomly selected as controls. Exclusion criteria included diabetes, chronic hypertension, severe heart, liver or renal dysfunction, other obstetric or medical syndromes and history of smoking, drinking, drug abuse or mental illness. There were no significant differences in the age, gravidity or delivery between patients with preeclampsia and controls (P>0.05). However, neonatal and placental weights significantly differed between the two groups (Table 1).

**Table 1:** Comparison of clinical data among groups.

	n	age(year)	gestational age (w)	neonatal weight(g)	placental weight (g)
control	15	28.22±4.98	38.01±5.36	3355.22±296.98	641.14±58.39
PE	45	29.63±5.56	37.51±5.62	3100.45±303.44**	585.42±60.23**
MPE	24	28.41±5.37	37.67±6.19	3143.31±315.75*	598.35±65.48*
SPE	21	30.35±6.01	36.88±5.21	2998.17±331.77**	576.21±68.36**

PE = preeclampsia. MPE = Moderate preeclampsia. SPE = Severe preeclampsia.

*P<0.05 compared with control group.

**P<0.01 compared with control group

### Sample collection

Four milliliters venous blood were collected from each individual following an overnight fast prior to preeclampsia treatment. After centrifugation for 15 minutes at 2500 r/min at 4°C, the supernatants were stored at −70°C until further analysis.

Two pieces of tissues were obtained immediately after delivery from the center of the maternal placental surface rather than the infarction and calcification area. The sample size was 1.0 cm × 1.0 cm × 1.0 cm. One sample was fixed with 10% paraformaldehyde for tissue microarray (TMA) preparation, and the other was stored at −80°C for real-time quantitative PCR (qRT-PCR) analysis.

### Enzyme-linked immunosorbent assay (ELISA) measurements

LXRα levels were measured with ELISA kits (DSL Co. Ltd, Shanghai, China). The sensitivity was 0.1 pg/mL. Endoglin levels were measured with ELISA kits (R&D Systems China Co. Ltd, Shanghai, China). The sensitivity was 0.01ng/mL. Operations were strictly carried out following up kit instructions.

### Tissue microarray (TMA) and immunohistochemistry analysis

TMA construction was performed as described previously [12]. Placental LXRα and endoglin expression was analyzed according to standard immunohistochemical methods [13]. The corresponding primary antibodies were purchased from Santa Cruz Biotechnology (Santa Cruz Biotechnology Inc., Santa Cruz, CA, USA; mouse anti-human LXRα, sc-377260, 1:100; mouse anti-human endoglin, sc-376381, 1:100). The staining intensity was graded [13] as follows: 0, no staining; 1, mild staining; 2, moderate staining; and 3, intense staining. Positive staining rate was scored as follows: 0, no staining of cells; 1, 1–25%; 2, 26–50%; and 3, > 50%. The sum of intensity and positive rate was designated as the staining score and graded as follows: 0–1 (-); 2 (+); 3–4 (++) and 5–6 (+++).

### qRT-PCR

Total RNA was isolated from placental tissues using TRIzol reagent (Invitrogen) according to the manufacturer’s instructions and reverse transcribed. qRT-PCR was performed with Universal SYBR Green PCR Master Mix using specific primers with the following sequences: LXRα forward 5′-TGGAGACATCTCGGAGGTAC-3′, LXRα reverse 5′-GCAATGAGCAAGGCAAACT-3′; Endoglin forward 5′-TAGCCCTGCGTCCCAAGA-3′, Endoglin reverse 5'-CGATGAGGAAGGCACCAAA-3'; GAPDH forward 5′-GAAGATGGTGATGGGATTTC-3′, GAPDH reverse 5′-GAAGGTGAAGGTCGGAGT-3′. The run conditions were: 94°C for 5 mins; 94°C for 30 seconds, 52°C for 30 seconds and 72°C for 40 seconds (30 cycles); and 72°C for 10 mins. Relative quantification was determined using the comparative CT method.

### Statistical analysis

All data analyses were performed using SPSS 13.0 software (SPSS Inc.,Chicago, IL, USA). Results were presented as the mean±SD. Clinical data and mean serum and placental gene levels of LXRα and endoglin among groups were compared by post-hoc pair-wise comparisons in ANOVA. Mean placental LXRα and endoglin protein expression levels were compared by the Mann–Whitney U test. Pearson and Spearman correlation coefficient analyses were performed to assess possible relationships. The receiver operating characteristic curve (ROC) analysis was performed to choose the best cut-off value for prediction and diagnosis of preeclampsia. P<0.05 was considered statistically significant.

## Results

### Comparison of serum LXRα and endoglin levels in preeclampsia group and control group

To assess associations between LXRα levels and clinical preeclampsia, serum LXRα levels were compared between patients with preeclampsia and normal pregnant women (control). As displayed in Table 2, LXRα levels in patients with preeclampsia were significantly higher than those in control group (P<0.05). Notably, LXRα levels in patients with severe preeclampsia were significantly higher than those in patients with moderate preeclampsia (P<0.05). The best cut-off value was 3.68 μg/ml by ROC analysis. There were 33 cases in which LXRα levels exceeded 3.68 μg/ml in the preeclampsia group but only 4 cases in the control group. The LXRα sensitivity, specificity and positive and negative predictive values were 66.00%, 80.00%, 89.19% and 48.48%, respectively.

**Table 2:** Comparison of LXRα levels in serum among groups.

	n	LXRα (μg/ml)
Control	15	2.88±0.54
PE	45	4.91±1.33*
MPE	24	4.27±0.47*
SPE	21	6.23±1.09**

PE = preeclampsia. MPE = Moderate preeclampsia. SPE = Severe preeclampsia.

*P<0.05 compared with control group.

** P<0.05 compared with MPE group.

As expected, serum endoglin levels were also significantly higher in patients with preeclampsia than those in control group (P<0.05). Moreover, endoglin levels were significantly higher in patients with severe preeclampsia than those in patients with moderate preeclampsia (Table 3). The best cut-off value was 14.66 ng/ml by ROC analysis. There were 31 cases in which endoglin levels exceeded 14.66 ng/ml in the preeclampsia group but only 3 cases in the control group. The endoglin sensitivity, specificity and positive and negative predictive values were 62.00%, 85.00%, 91.18% and 47.22%, respectively.

**Table 3:** Comparison of endoglin levels in serum among groups.

	n	Endoglin (ng/ml)
Control	15	11.18±4.33
PE	45	29.12±6.24*
MPE	24	27.59±7.84*
SPE	21	38.25±9.04**

PE = preeclampsia. MPE = Moderate preeclampsia. SPE = Severe preeclampsia.

*P<0.05 compared with control group.

** P<0.05 compared with MPE group.

### Placental LXRα and endoglin protein expression in preeclampsia group and control group

**Fig 1:**
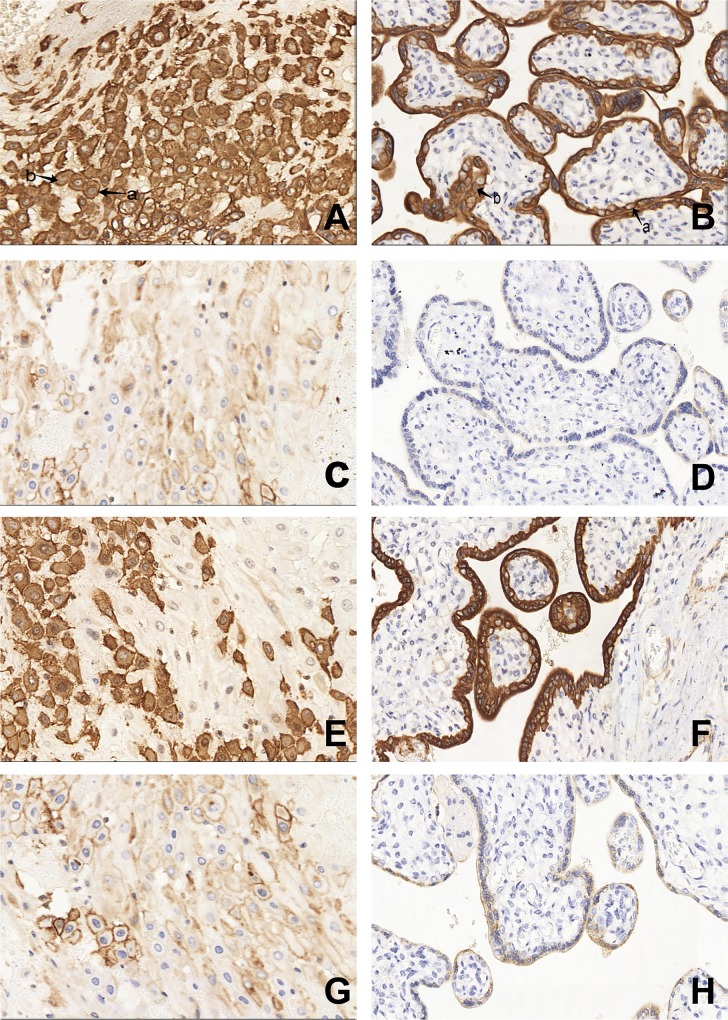
TMA samples preparation and immunohistochemical staining of LXRα and endoglin expression in decidual cells and trophoblast cells from placentaes of preeclampsia. Expression of LXRα in preeclampsia decidual tissue (a membrane, b cytoplasm). (B) Expression of LXRα in preeclampsia villous trophoblastic tissue (a membrane, b cytoplasm). (C) Expression of LXRα in normal decidual tissue. (D) Expression of LXRα in normal villous trophoblastic tissue. (E) Expression of endoglin in preeclampsia decidual tissue. (F) Expression of endoglin in preeclampsia villous trophoblastic tissue. (G) Expression of endoglin in normal decidual tissue. (H) Expression of endoglin in normal villous trophoblastic tissue. Original magnication X400

To confirm these findings in the placenta, a tissue microarray and immunohistochemistry were performed to assess LXRα expression in patients with preeclampsia and normal controls. The LXRα protein was localized to the membrane and cytoplasm of decidual cells and villous trophoblasts in the placentas of both patients with preeclampsia and normal controls (Fig 1A–1D). Importantly, placental LXRα levels in patients with preeclampsia were significantly higher than those of controls (P<0.05). Moreover, patients with severe preeclampsia also displayed higher LXRα expression than that of patients with moderate preeclampsia (P<0.05) (Table 4). Immunohistochemical analysis indicated that endoglin was expressed in the membrane and cytoplasm of placental decidual cells and villous trophoblasts (Fig 1E–1H). As expected, placental endoglin levels were positively associated with disease activity (Table 5).

**Table 4:** Comparation of LXRα expression intensity in placenta among groups.

	n	LXRα				
		-	+	++	+++	positive rate (%)
Control	15	10	2	2	1	33.33
PE	45	8	12	12	13	82.22 ^*^
MPE	24	7	6	5	6	70.83 ^*^
SPE	21	1	6	7	7	95.24^**^

PE = preeclampsia. MPE = Moderate preeclampsia. SPE = Severe preeclampsia.

*P<0.05 compared with control group.

** P<0.05 compared with MPE group.

**Table 5:** Comparation of Endoglin expression intensity in placenta among groups.

	n	Endoglin				
		-	+	++	+++	positive rate (%)
Control	15	12	1	1	1	20.00
PE	45	13	11	11	10	71.11^*^
MPE	24	10	6	5	3	58.33^*^
SPE	21	3	5	6	7	85.71^**^

PE = preeclampsia. MPE = Moderate preeclampsia. SPE = Severe preeclampsia.

*P<0.05 compared with control group.

**P<0.05 compared with MPE group.

### Placental LXRα and endoglin gene expression in preeclampsia group and control group

**Fig 2:**
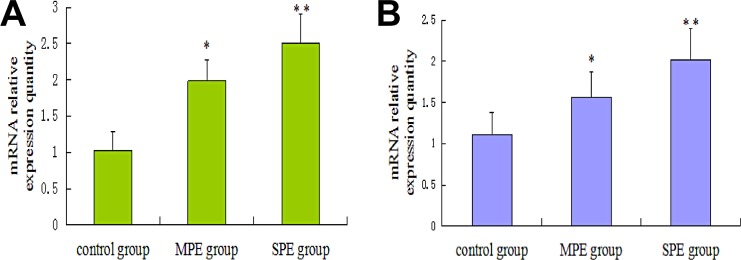
Real-time quantitative PCR analysis of LXRα and Endoglin levels in each group of placentas. (A) LXRα mRNA level was examined by qRT-PCR in each group of placentas. (B) Endoglin mRNA level was examined by qRT-PCR in each group of placentas. MPE = Moderate preeclampsia. SPE = Severe preeclampsia. *P<0.05 compared with control group. **P<0.05 compared with MPE group.

We next compared placental LXRα and endoglin mRNA levels by qRT-PCR. As displayed in Fig 2A and 2B, LXRα and endoglin expression in patients with preeclampsia was significantly higher than that in normal controls, and this increase was more significant in patients with severe preeclampsia. Taken together, these results suggested that placental LXRα and endoglin levels were upregulated in patients with preeclampsia and closely associated with preeclampsia disease activity.

### Correlations between LXRα and endoglin levels in serum and placenta

LXRα and endoglin levels in serum and placenta from patients with preeclampsia were positively correlated (r = 0.486, P<0.01; r = 0.569, P<0.01). Additionally, LXRα and endoglin levels in serum and placenta from the control group were also positively correlated (r = 0.493, P<0.01; r = 0.475, P<0.01).

## Discussion

Preeclampsia is characterized by impaired function and disturbance of lipid metabolism, including reduced high density lipoprotein cholesterol and apolipoproteins A1 and B, which are protective for blood vessels, and increased total cholesterol, triglycerides, low density lipoprotein cholesterol and very low density lipoprotein cholesterol, which are destructive to blood vessels. Therefore, it is possible that lipid metabolism disorders may lead to preeclampsia development. Evidence suggests that LXRα is highly expressed in the liver, adipose tissue, kidney and spleen and is associated with abnormal lipid metabolism [14]. LXRα is a key regulator of placental lipid metabolism in pregnancy. It participates in cholesterol decomposition, absorption and transport and inhibits HCG secretion in trophoblasts [15]. However, the role of LXRα in preeclampsia remains unknown.

To the best of our knowledge, this is the first report comparing LXRα levels between patients with preeclampsia and normal pregnant women. Placental LXRα protein and mRNA levels were significantly higher in patients with preeclampsia than those in control subjects, and the increase was positively associated with disease severity. Thus, our data indicated that changes in LXRα levels might be closely related to preeclampsia development and progression. A number of studies have investigated the potential regulatory mechanisms underlying this observation. Specifically, Aye et al. [16] determined that LXRα suppressed trophoblast invasion and differentiation, thereby resulting in shallow placental implantation and preeclampsia development. Additionally, LXRα induced liver sterol regulatory element binding protein-1c expression [17,18], which caused an increase in liver and plasma triglycerides, free fatty acids and other peroxidase substrates. Subsequently, the following changes occur: lipid metabolism disorders, vascular endothelial cell damage, acute atherosclerosis of small arteries in the uterus and placenta and diminished placental function. Ultimately, preeclampsia results in severe complications, including fetal growth restriction and fetal intrauterine distress. Furthermore, LXRα activated the peroxisomal proliferator-activated receptor gamma retinoid X receptor pathway by upregulating low density lipoprotein production, producing toxic injury to trophoblasts and inhibiting trophoblast invasion, which resulted in disability of uterine spiral artery recasting and preeclampsia development [19]. Finally, LXRα enhanced macrophage TNF mRNA levels to induce local inflammation [18], thereby causing apoptosis of endothelial and smooth muscle cells, T cell invasion and local tissue necrosis. Moreover, TNF induced production of prostaglandin and endothelin, which are strong vasoconstrictors, in vascular endothelial cells and caused vascular contraction and blood pressure elevation. These are only possible mechanisms, and a biological role of LXRα in preeclampsia warrants further investigation.

Another finding of this study was that serum LXRα levels in patients with preeclampsia were also significantly higher than controls, and those from severe individuals were higher than those from patients with moderate preeclampsia. Thus, serum LXRα levels may be tested to monitor the occurrence and severity of preeclampsia. Additionally, the sensitivity and specificity of LXRα were 66.00% and 80.00%, respectively, indicating that LXRα measurements provided high sensitivity and specificity for established preeclampsia diagnosis and may be used as a novel predictor for clinical application.

Endoglin, part of the TGF-β receptor complex, was predominately expressed on vascular endothelial cells and involved in TGF-β and TGF-β receptor signaling. By antagonizing the inhibitory effects of TGF-β1 on vascular endothelial cell proliferation, endoglin can promote angiogenesis and is a biomarker of endothelial cell proliferation. Endoglin expression is significantly upregulated during pregnancy and is rapidly reduced after delivery, indicating that the placenta may be the primary source of endoglin during pregnancy [20].

Recent studies [20,21] demonstrated that serum endoglin concentrations were significantly increased in patients with preeclampsia and positively associated with preeclampsia severity. Tarek et al. [22] reported that serum endoglin levels were significantly higher in pregnant women with high-risk preeclampsia than in healthy pregnant women at gestational week 13. Additionally, endoglin levels were significantly higher in patients with early-onset preeclampsia than those in patients with late-onset preeclampsia. Furthermore, soluble endoglin levels were positively correlated with blood pressure as well as urinary proteins. Our study demonstrated dramatically higher serum endoglin levels in the preeclampsia group compared with those in the control group, and this increase was strongly positively correlated with disease severity. Interestingly, these results were in agreement with those of Stepan et al. [23]. Additionally, the endoglin sensitivity, specificity, and positive and negative predictive values were 62.00%, 85.00%, 91.18% and 47.22%, respectively (The PPV and NPV would be very different in an unselected population of pregnant women). Collectively, these results indicated that serum endoglin concentration was useful for predicting preeclampsia and may be a new forecast index for clinical application.

Our study determined that placental endoglin protein and mRNA levels in patients with preeclampsia were distinctly higher than those in normal controls. This difference was more apparent in individuals with severe preeclampsia compared with that in individuals with moderate preeclampsia. Therefore, our data indicated that increased endoglin was associated with preeclampsia development and progression. Venkatesha et al. [20] found that the permeability of organs, including liver, lung and kidney, was significantly increased when mice were injected with an adenovirus expressing soluble endoglin. Their findings suggested that overexpression of soluble endoglin can enhance vascular permeability and reduce placental perfusion by damaging the integrity of endothelial cells. Recent studies have indicated that endoglin can inhibit NO and heme oxygenase-1 (HO-1) synthesis and reduce production of CO and bilirubin, thereby leading to endothelial dysfunction, blood pressure elevation and development of severe preeclampsia [24–26].

Henry-Berger et al. [10] reported that endoglin was highly expressed in choriocarcinoma JAR cells following stimulation with LXRα activators. They demonstrated that LXRα binds the endoglin promoter and mediates endoglin activation. We determined that LXRα expression positively correlated with endoglin expression in serum and placenta from patients with preeclampsia. These results indicate that increased LXRα might inhibit the proliferation, infiltration and migration ability of trophoblasts by upregulating endoglin production and thus participating in preeclampsia development.

In conclusion, LXRα and endoglin levels may be involved in preeclampsia development and progression and may be used as biomarkers for clinical application. In the future, effective suppression of placental LXRα and endoglin expression or neutralization of LXRα and endoglin activation in the circulation may provide new methods to prevent and treat preeclampsia. Future studies will address the following: 1) analyze early-stage pregnant women and confirm that LXRα abnormalities occur prior to established disease; 2) expand the number and diversity of subjects; 3) measure these in a more thorough prospective study where LXRα, potentially in conjunction with endoglin of later severe PE, to determine if it can identify future disease. Further research is currently under investigation in our laboratory.
